# Beyond artificial academic debates: for a diverse, inclusive, and impactful ethnobiology and ethnomedicine

**DOI:** 10.1186/s13002-023-00611-6

**Published:** 2023-09-08

**Authors:** Victoria Reyes-García

**Affiliations:** 1 Institució Catalana de Recerca i Estudis Avançats (ICREA), 08010 Barcelona, Spain; 2 Institut de Ciència i Tecnologia Ambientals, Universitat Autònoma de Barcelona (ICTA-UAB), Cerdanyola del Vallès, 08193 Barcelona, Spain; 3 Departament d’Antropologia Social i Cultural, Universitat Autònoma de Barcelona, Cerdanyola del Vallès, 08193 Barcelona, Spain

**Keywords:** Biological indices, Co-production of knowledge, Epistemology, Indigenous and local knowledge, Methods

## Abstract

In answer to the question *“Should ethnobiology and ethnomedicine more decisively foster hypothesis-driven forefront research able to turn findings into policy and abandon more classical folkloric studies?*”, in this essay I argue that a major strength of ethnobiology and ethnomedicine is their ability to bridge theories and methods from the natural sciences, the social sciences, and the humanities. Hypothesis-driven research is a powerful way to structure thinking that can lead to forefront research findings. But hypothesis-driven research is not the only way to structure thinking and is not a necessary condition to impact policymaking. To increase policy impact, ethnobiology and ethnomedicine should continue nurturing a mixture of complementary methods and inclusive approaches as fragmentation through opposing different approaches might weaken the discipline. Moreover, with the aim to play a fundamental role in building bridges between different knowledge systems and co-producing solutions towards sustainability, the discipline could benefit from enlarging its epistemological grounds through more collaborative research. Ethnobiologists' research findings, hypothesis-driven, descriptive, or co-constructed can become leverage points to transform knowledge into actionable outcomes in different levels of decision-making.

## Introduction

Researchers in ethnobiology and ethnomedicine debate the appropriate use of different methodologies and epistemologies [1, 2], with repeated calls for engaging in theory-inspired and hypothesis-driven research [3]. The debate has been framed both in terms of the merits of different approaches [1, 2] and in terms of the development of the discipline [4]. Indeed, departing from its descriptive origins, and as data accumulate, theory-inspired and hypothesis-driven approaches to study why and how local people relate to different elements of the environment have grown [1, 4]. Ethnobiologists’ toolbox to conduct hypothesis-driven research has grown in parallel and now includes tools such as generalized linear modelling, structural equation modelling, phylogenetic generalized least squares, social network analysis, species distribution, and predictive modelling [1]. Despite this growth, descriptive approaches continue to be popular in ethnobiology and ethnomedicine. Moreover, as researchers in the field come from different epistemological and methodological backgrounds, the question of whether hypothesis-driven should replace descriptive approaches remains, as exemplified in the question set in this initial Debate Series on the *Journal of Ethnobiology and Ethnomedicine.*

To answer the question posed for debate (i.e., *“Should ethnobiology and ethnomedicine more decisively foster hypothesis-driven forefront research able to turn findings into policy and abandon more classical folkloric studies?*”), in this opinion piece, I examine the three components of the question independently.

## Should ethnobiology and ethnomedicine foster hypothesis-driven forefront research?

Formulating hypotheses is a very powerful and potentially efficient way of structuring thinking. Hypotheses testing often leads to novel findings that advance our understanding of people–environments interactions. For example, the use of indices derived from ecological research in ethnobotany, first proposed by Begossi [5], has allowed testing hypotheses on the relative importance of different plant species according to different criteria and has been used to improve ethnopharmacological screening through testing the efficacy of medicinal plants [6]. Hypothesis-driven research allows to test ideas on the distribution of reports of climate change impacts on local social–ecological systems [7] or on the medicinal relevance of species across cultural groups [8]. Results from hypothesis-driven research sometimes help challenge assumptions on the relations between people and plants. For example, in recent work, Mateo and colleagues use the most comprehensive dataset of traditional uses of plants in Spain to show that gathering medicinal plants for self-consumption does not lead to overharvesting [9]. By testing the connections between the cultural importance of medicinal vascular plants traditionally used in Spain for self-treatment and their availability, conservation, and legal protection status, the authors are able to show that—contrary to what is commonly assumed—most medicinal plant species used for self-treatment in Spain are common, readily available, and not threatened, which suggests that the use of medicinal plants for domestic purposes only does not result in overexploitation [9].

In sum, hypothesis-driven research in ethnobiology and ethnomedicine allows for understanding hidden patterns in data. Results from hypothesis-driven studies are also valuable to communicate with natural scientists, as they share similar epistemological and methodological backgrounds. However, whether and how results from hypothesis-driven research contribute to policy action is a question that should be analysed separately.

## Should ethnobiology and ethnomedicine foster research able to turn findings into policy?

Recent years have seen a global effort to include research results into global policy making, as seen in the works of the Intergovernmental Panel on Climate Change (IPCC) and the Intergovernmental Platform on Biodiversity and Ecosystems Services (IPBES) [10, 11]. While these initiatives do not remain free from power imbalances and disciplinary biases towards natural sciences and scientific epistemologies [11], they make a conscious effort to bring together natural and social scientists and to incorporate evidence from plural knowledge systems, with particular emphasis on Indigenous and local knowledge systems [12, 13].

Results from these experiences suggest that governance, planning and decision-making at local, regional, national, and international levels generally continues to overlook the ecological knowledge and associated worldviews and values and beliefs of Indigenous Peoples and local communities [14–16]. These experiences also suggest that successfully bringing Indigenous and local knowledge into policy arenas requires a deliberate effort from researchers and policymakers to create an approach that facilitates recognition of different knowledge systems, identifies questions relevant for different actors at various scales, mobilizes funding, and recognizes the extra time required to engage networks of stakeholders with diverse worldviews [12]. Moreover, because policy debates often entail epistemological differences, attempts to address them should acknowledge these differences. For example, attempts to protect Indigenous and local knowledge systems are largely treated as a matter of intellectual property rights [17]. However, the regulatory framework of intellectual property rights does not necessarily fit with how Indigenous Peoples and local communities manage their knowledge [18]. Consequently, aiming to contribute to this policy debate using results from hypothesis-driven research might—in fact—reinforce the epistemological divide, thus not really contributing to advance the discussion and bring about political change.

All in all, researchers, funding agencies, and global science organizations suggest that research aiming at addressing the complex nature of contemporary challenges is most effective when co-produced by different actors [19]. Because they hold knowledge essential for setting realistic and effective biodiversity targets, have conceptualizations of nature that can contribute to the vision of "living in harmony with nature," and have rights to essential territories and resources, Indigenous Peoples and local communities need more recognition in policymaking [20]. Given its research interests, ethnobiology and ethnomedicine are well positioned to engage in the co-production of knowledge with Indigenous Peoples and local communities. Moreover, ethnobiology and ethnomedicine should aspire to go beyond policymaking and aim to have an impact in other levels of decision-making at local, regional, national, and international levels.

## Should ethnobiology and ethnomedicine abandon classic folkloric studies?

It is easy to argue that descriptive studies in ethnobiology and ethnomedicine continue to be needed. Descriptive studies provide the data needed for hypothesis-driven research. For example, the Spanish inventory of traditional knowledge is the largest compilation of descriptions of plant and animal uses in different locations in Spain [21]. Efforts to compile and organize such descriptive work have set the bases to test hypotheses, as in the work mentioned above [9]. Descriptive studies are also important to provide the needed context to interpret hypothesis-driven research results. For example, in a critical analysis of indices used in ethnobiology, Leonti [2] argues that it is unlikely that a simple number can summarize the cultural value or importance of a species, for which descriptive information provides the needed context to understand and correctly interpret results.

Beyond this utilitarian view of descriptive data to support quantitative analysis, three other important arguments support the need for in-depth descriptive work. First, descriptive work can lead to important insights in its own. For example, the recent IPBES Values Assessment emphasizes the importance of integrating relational values, or the types of relationships between humans and nature, such as care, social bonding, place attachment, and spiritual meanings in biodiversity policy. Research in this topic comes from qualitative analyses of the different ways people value nature, and not from hypothesis-driven work, but can become an important leverage point in current biodiversity policies [22]. Second, descriptive work offers a good basis to enable effective empowering dialogues among different stakeholders, a key element for integrated sustainability science [4]. Exclusively focusing on results from hypothesis-driven research might lead to organize policy debates in non-inclusive ways, thus perpetuating power imbalances between knowledge systems. Adding results from descriptive research might help leverage the discussion field between stakeholders coming from different epistemologies. Finally, results from descriptive research can be translated into illustrated field guides (e.g., on local plant uses), which have a much wider audience than scientists, also reaching policy makers, laypeople, students, teachers, Indigenous Peoples and local communities, who can use them locally (e.g., in school education, ecotourism, or as a repository of local knowledge). As, in many parts of the world, such information is otherwise not available, or not of sufficient quality, descriptive studies in ethnobiology and ethnomedicine can also be locally important.

In sum, abandoning descriptive work would not only close windows to poorly known worlds, thus impoverishing the basis on which we can develop and test hypotheses, but it might also contribute to maintain barriers that hinder dialogue between knowledge systems. As mentioned, formulating and testing theory-driven hypotheses is a powerful way to structure thinking, but restricting knowledge production to only one way of thinking and the assumptions that come with it might simply result in an impoverished view of the world.

## Connecting the dots as a way to conclude

In this essay, I have separately analysed the three subquestions that constitute the original question posed for debate. I have argued that, while hypothesis-driven research might lead to forefront research findings, this is not necessarily a condition to impact policymaking. I have also argued that recent experiences show that a productive way to impact policymaking is to include the views and values of different stakeholders through empowering dialogues. This implies the ability to generate and communicate knowledge to a wide range of stakeholders to leverage the field for dialogue.

As an interdisciplinary field of research, ethnobiology and ethnomedicine draw on theories and methods from the natural sciences, the social sciences, and the humanities. Here lies its strength. To increase its policy impact, ethnobiology and ethnomedicine should continue nurturing a mixture of complementary methods and inclusive approaches, as fragmentation through opposing different approaches might weaken the discipline [23]. Moreover, ethnobiology and ethnomedicine could also increase the focus on process. Attention to the underlying assumptions of the ways in which we generate hypotheses, collect data, test ideas, represent people, and use data are important, but often neglected topics of enquiry. Finally, with the aim to play a fundamental role in building bridges between a range of knowledges and co-producing solutions towards sustainability [19], ethnobiology and ethnomedicine could benefit from enlarging its epistemological grounds through more collaborative research oriented to the co-production of knowledge.

To be policy impactful, ethnobiology and ethnomedicine should continue to build on diverse theoretical and methodological background and increase efforts to include a range of actors, particularly Indigenous Peoples and local communities, in empowering dialogues for the co-production of new knowledge. Ethnobiologists research findings, hypothesis-driven, descriptive, or co-constructed can become leverage points to transform knowledge into actionable outcomes in different levels of decision-making.

## Data Availability

Not applicable.

## Abbreviations

IPBES: Intergovernmental Platform on Biodiversity and Ecosystems Services
IPCC: Intergovernmental Panel on Climate Change

## References

[1] Gaoue OG, Moutouama JK, Coe MA, Bond MO, Green E, Sero NB, et al. Methodological advances for hypothesis-driven ethnobiology. Biol Rev. 2021;96:2281–303. 10.1111/brv.12752. 34056816 10.1111/brv.12752

[2] Leonti M. The relevance of quantitative ethnobotanical indices for ethnopharmacology and ethnobotany. J Ethnopharmacol. 2022;288:115008. 35066067 10.1016/j.jep.2022.115008

[3] Gaoue OG, Coe MA, Bond M, Hart G, Seyler BC, McMillen H. Theories and major hypotheses in ethnobotany. Econ Bot. 2017;71:269–87. 10.1007/s12231-017-9389-8.

[4] Vibrans H, Casas A, Vibrans H, Casas A. Roads traveled and roads ahead: the consolidation of Mexican ethnobotany in the new millennium an essay. Bot Sci. 2022;100:263–89.

[5] Begossi A. Use of ecological methods in ethnobotany: diversity indices. Econ Bot. 1996;50:280–9. 10.1007/BF02907333.

[6] Atanasov AG, Zotchev SB, Dirsch VM, Orhan IE, Banach M, Rollinger JM, et al. Natural products in drug discovery: advances and opportunities. Nat Rev Drug Discov. 2021;20:200–16. 33510482 10.1038/s41573-020-00114-z PMC7841765

[7] Reyes-García V, Álvarez-Fernández S, Benyei P, García-Del-Amo D, Junqueira AB, Labeyrie V, Li X, Porcher V, Porcuna-Ferrer A, Schlingmann A, Soleymani R. Local indicators of climate change impacts described by indigenous peoples and local communities: Study protocol. PLoS One. 2023 Jan 5;18(1):e0279847. 10.1371/journal.pone.0279847. 10.1371/journal.pone.0279847 PMC9815565 36602984

[8] Heinrich M, Ankli A, Frei B, Weimann C, Sticher O. Medicinal plants in Mexico: healers’ consensus and cultural importance. Soc Sci Med. 1998;47:1859–71. 9877354 10.1016/s0277-9536(98)00181-6

[9] Mateo-Martin J, Benitez J, Gras A, Molina M, Reyes-García V, Tardío J, et al. Cultural importance, availability and conservation status of Spanish wild medicinal plants: implications for sustainability. People Nat. 2023. 10.1002/pan3.10511.

[10] Watson RT. Turning science into policy: challenges and experiences from the science–policy interface. Philos Trans R Soc B: Biol Sci. 2005;360:471. 10.1098/rstb.2004.1601 PMC1569452 15814358

[11] Balvanera P, Jacobs S, Nagendra H, O’Farrell P, Bridgewater P, Crouzat E, et al. The science-policy interface on ecosystems and people: challenges and opportunities. Ecosyst People. 2020;16:345–53. 10.1080/26395916.2020.1819426.

[12] McElwee P, Fernández-Llamazares Á, Aumeeruddy-Thomas Y, Babai D, Bates P, Galvin K, et al. Working with Indigenous and local knowledge in large-scale ecological assessments: reviewing the experience of the IPBES global assessment. J Appl Ecol. 2020;57:1666–76. 10.1111/1365-2664.13705.

[13] Hill R, Adem Ç, Alangui WV, Molnár Z, Aumeeruddy-Thomas Y, Bridgewater P, et al. Working with indigenous, local and scientific knowledge in assessments of nature and nature’s linkages with people. Curr Opin Environ Sustain. 2020;43:8–20.

[14] Turner NJ, Cuerrier A, Joseph L. Well grounded: indigenous peoples’ knowledge, ethnobiology and sustainability. People Nat. 2022;4:627–51. 10.1002/pan3.10321.

[15] Ford JD, Cameron L, Rubis J, Maillet M, Nakashima D, Willox AC, et al. Including indigenous knowledge and experience in IPCC assessment reports. Nat Clim Chang. 2016;6:349–53.

[16] Carmona R. Global guidelines, local interpretations: ethnography of climate policy implementation in Mapuche territory. Southern Chile Clim Policy. 2023. 10.1080/14693062.2023.2194267.

[17] Golan J, Athayde S, Olson EA, McAlvay A. Intellectual property rights and ethnobiology: an update on posey’s call to action. J Ethnobiol. 2019;39:90–109.

[18] Reyes-García V, Tofighi-Niaki A, Austin BJ, Benyei P, Danielsen F, Fernández-Llamazares Á, et al. Data sovereignty in community-based environmental monitoring: toward equitable environmental data governance. Bioscience. 2022;72:714. 35923191 10.1093/biosci/biac048 PMC9343228

[19] Norström AV, Cvitanovic C, Löf MF, West S, Wyborn C, Balvanera P, et al. Principles for knowledge co-production in sustainability research. Nat Sustain. 2020;3:182–90.

[20] Reyes-García V, Fernández-Llamazares Á, Aumeeruddy-Thomas Y, Benyei P, Bussmann RW, Diamond SK, et al. Recognizing Indigenous peoples’ and local communities’ rights and agency in the post-2020 biodiversity agenda. Ambio. 2022;51:84–92. 10.1007/s13280-021-01561-7. 34008095 10.1007/s13280-021-01561-7 PMC8651947

[21] Pardo-de-Santayana M, Morales R, Aceituno-Mata L, Molina M. Inventario Español de conocimientos tradicionales relativos a la biodiversidad. Madrid: Ministerio de Agricultura y Pesca, Alimentación y Medio Ambiente; 2014.

[22] IPBES. Summary for policymakers of the methodological assessment of the diverse values and valuation of nature of the intergovernmental science-policy platform on biodiversity and ecosystem services (IPBES). 2022. https://zenodo.org/record/7410287.

[23] Albuquerque UP, Ludwig D, Feitosa IS, de Moura JMB, de Medeiros PM, Gonçalves PHS, et al. Addressing social-ecological systems across temporal and spatial scales: a conceptual synthesis for ethnobiology. Hum Ecol. 2020;48:557–71. 10.1007/s10745-020-00189-7.

